# Effect of Residual Stresses on Fatigue Crack Growth: A Numerical Study Based on Cumulative Plastic Strain at the Crack Tip

**DOI:** 10.3390/ma15062156

**Published:** 2022-03-15

**Authors:** Diogo M. Neto, Micael F. Borges, Edmundo R. Sérgio, Fernando V. Antunes

**Affiliations:** Centre for Mechanical Engineering, Materials and Processes (CEMMPRE), Department of Mechanical Engineering, University of Coimbra, 3030-788 Coimbra, Portugal; diogo.neto@dem.uc.pt (D.M.N.); micaelborges96@gmail.com (M.F.B.); dudasergio98@gmail.com (E.R.S.)

**Keywords:** fatigue crack growth, residual stresses, crack closure, crack tip plastic strain

## Abstract

Residual stresses affect the fatigue behavior, given that compressive stresses delay the phenomenon, while tensile stresses accelerate it. However, the mechanisms behind the effect of residual stresses are not totally understood. A numerical study is developed here to understand the effect of thermal residual stresses (TRSs) on fatigue crack growth (FCG). The crack driving force was assumed to be the cumulative plastic strain at the crack tip. The heating of a region ahead of the crack tip produced elastic compressive TRS, which were 69% of material’s yield stress. Alternatively, plastic deformation was produced by severe cooling followed by heating to generate compressive residual stresses. The crack propagation in the compressive residual stress field produced a decrease in the FCG rate. On the other hand, without the contact of crack flanks, the TRS showed no effect on FCG. Therefore, the TRSs only affect FCG by changing the crack closure level.

## 1. Introduction

Residual stresses affect fatigue behavior; therefore, the definition of inspection intervals of critical components must include their effect. It is well known that compressive stresses delay fatigue damage, while tensile stresses accelerate the phenomenon. The residual stresses may be introduced unintentionally by the technological process, namely by quenching, welding, casting, or additive manufacturing. On the other hand, compressive stresses may be deliberated produced using shot peening [1], hammer peening, indentation [2], laser shock peening [3], cold expansion, overloading, bending plastic deformation [4], or surface mechanical attrition treatment (SMAT) [5]. Farrahi et al. [6] used shot peening and the indentation technique, and observed that the maximum value of residual stress was on the surface in both cases. However, the residual stress field induced by the indentation technique, which involves the application of local compression into the specimen at the crack tip or at both sides of the expected crack path, was deeper. A punching tool or a steel ball can be used to make an indentation. With the use of shot peening, Wang et al. [1] obtained residual stresses up to a depth of about 400 μm. The residual stresses at surface and the maximum residual stresses increased linearly with material’s yield stress and ultimate strength, respectively. SMAT is similar to shot-peening; however, the size of the balls is higher and the balls impact the specimen at random angles. Laser shock peening (LSP) is another life improvement process which is based on the radiation of a laser beam at a very short time (several nanoseconds) on the material surface [3]. Shock waves are generated and penetrated into the material causing plastic deformation. LSP is very effective when dealing with thick components [7], while for thin panels, typical of aerospace structures, the stress distribution can be detrimental for FCG [8,9]. Cold working of holes is widely used in aeronautical industry to induce residual stresses [10,11,12]. A mandrel of slightly larger diameter than the original hole diameter is inserted, plastically deforming the material concentrically around the hole. The application of overloads also changes the residual stresses ahead of the crack tip, producing retardation. Typically, the effect of surface treatments (shot peening, hammer peening, laser shock peening, or SMAT) extends over less than 1 mm [3,5,13]. On the other hand, the cold working of holes may extend over several millimeters [14]. In relatively thick specimens, the surface treatment may produce a significant tunnelling of the crack front because the surface region has compressive residual stresses while the interior regions have tensile stresses.

According Lacarac et al. [10], marked improvements in fatigue life may be produced by reductions in the fatigue crack growth (FCG) rate. The improvement in fatigue behavior of the component is usually attributed to (i) the strain hardening of surface layers which increases the yield stress of the material; (ii) the compressive residual stresses in surface layers; and (iii) the final surface finish quality and structural changes [13]. The redistribution of residual stresses resulting from the crack propagation can play an important role, leading to the development of different modes of crack growth (i.e., with and without crack closure) [4]. However, the effect of residual stress fields on FCG and fatigue life is not totally understood, which is important for its incorporation into the mechanical design process of engineering components.

The estimation of fatigue life, including the effect of residual stresses, has been performed using different approaches. Superposition techniques are often used when assessing the effects of a known residual stress field on fatigue crack propagation [4,15]. The superposition involves the computation of a stress intensity factor which is associated with the initial pre-existing residual stress field, *K_R_*. This stress intensity factor is added to *K* value due to external loads. The stress intensity factor range Δ*K* is independent of the residual stress, and only the stress ratio *R* is affected. Negative resultant *K* values may be set to zero to simulate a closed crack [15,16]. However, the validity of *K* is not questioned in these approaches and the redistribution of stresses resulting from the crack growth is not included in the analysis. In fact, there is an intense debate in the literature between the supporters of the elastic superposition method [17] and those who question its applicability for the prediction of the FCG [18,19]. An alternative approach uses crack closure values to define an effective Δ*K*. LaRue and Daniewicz [15] predicted crack closure values in a residual field produced by overloading of a specimen with hole. The *da/dN-*Δ*K_eff_* curves needed to predict fatigue life are obtained at a high stress ratio. Newman and Daniewicz [20] used the FASTRAN life prediction code to predict FCG in specimens with compressive residual stresses, including the effect of crack closure. The residual stresses were produced by remote overloading and cold-expansion. The approach based on crack closure is interesting; however, there is no consensus about the quantification of the crack closure level. A comparison of the conventional closure measurements from the round robin tests organized by the authorized ASTM Task Group E24.04.04 on the same material and specimen geometry has indicated serious inconsistencies depending on the laboratory, investigator, and technique used. One of the conclusions drawn from this work was that “scatter of this magnitude would make it very difficult to develop a clear picture of closure effects and to verify quantitative models of closure effects using data from the literature” [21]. Besides, the load range below the opening load may also contribute to FCG [22].

However, *K*-based approaches are crucially limited, as they do not help to understand the mechanisms acting at the crack tip which are responsible for FCG. Therefore, alternative approaches based on non-linear parameters have been proposed, i.e., the force approach has been replaced by deformation and energy approaches. The non-linear parameters used were the plastic CTOD [23], the dissipated energy [24], J integral [25], crack tip plastic zones [26], and cyclic plastic strain [27]. FCG may be predicted numerically using these parameters. However, although there are good simulations of the residual stresses induced by different technological processes [2,3,4,11,28], the prediction of the FCG rate considering non-linear parameters and the residual stresses is not frequent. The nonlinear viscoelastic constitutive theory developed by Thamburaja et al. [29] uses a non-local description to describe the deformation and fracture of viscoelastic solids. The results show that this computational approach can reproduce the same response obtained using the extended finite element method in viscoelastic solids undergoing fracture [30]. Recently, Mozafari et al. [31] developed a small-strain microplasticity-based model to predict the fatigue behavior of metals, which helps to capture the inelastic work dissipation due to microplasticity. This model was successfully applied to predict the fatigue life under variable amplitude loading conditions [32], as well as the prediction of multiaxial fatigue with proportional and non-proportional loading [33]. The results show that this approach can describe the experimental data better than the current empirical approaches. A numerical approach based on cumulative plastic strain has been used by the authors to predict FCG [22]. The crack tip node is released when this cumulative strain reaches a critical value, which is calibrated using one experimental value of *da/dN*. This model includes the effects of plasticity-induced crack closure, residual stresses, partial closure, crack tip blunting, and material hardening. On the other hand, roughness-induced and oxide-induced crack closure, which are more relevant near-threshold, are not modeled. This numerical prediction of FCG, based on crack tip cumulative plastic strain, has a high level of maturity which has been proven in previous studies. In fact, the approach was able to qualitatively predict the effects of Δ*K* [34], stress ratio [35], stress state [36], overloads [22,35], and overload ratio [35]. Besides, the direct comparisons made with experimental results validated the assumption that cyclic plastic deformation is the main crack driving force. Borges et al. [37] successfully predicted the effect of Δ*K* observed experimentally in 2024-T251 aluminum alloy and 18Ni300 steel, while Neto et al. [35] predicted the effect of stress ratio, and Neto et al. [38] predicted the effect of Superblock2020 load pattern. Therefore, this approach can be applied with confidence to predict FCG rates in new situations. The main objective of this work is to understand the mechanisms behind the effect of residual stresses on FCG. More specifically, it is intended to check: (i) if crack closure is the only mechanism behind the effect of residual stresses; and (ii) if there is a difference between elastic residual stresses and residual stresses induced by plastic deformation. The elastic residual stress field is introduced by heating the material at a region ahead of the initial crack tip position. Alternatively, plasticity is induced by locally cooling the material to a relatively low temperature followed by heating to produce compressive residual stresses. Note that the proposed studies are not easily replicable experimentally, which is not a problem considering the recognized robustness of the numerical approach. The link between residual stresses and crack closure is established comparing predictions obtained with and without the contact of the crack flanks. The elimination of the contact of crack flanks was attempted in the experimental work of Vor et al. [39]; however, the curved shape of the crack front and the concentration of crack closure in a very small region immediately behind the crack front made this impossible. Furthermore, the inclusion of elastic residual stresses by local heating is not easy to realize experimentally because heat conduction in metals quickly attenuates the residual stresses, though this can be numerically worked out by artificially decreasing material thermal conductivity.

## 2. Numerical Model

### 2.1. Geometry and Boundary Conditions

**Figure 1:**
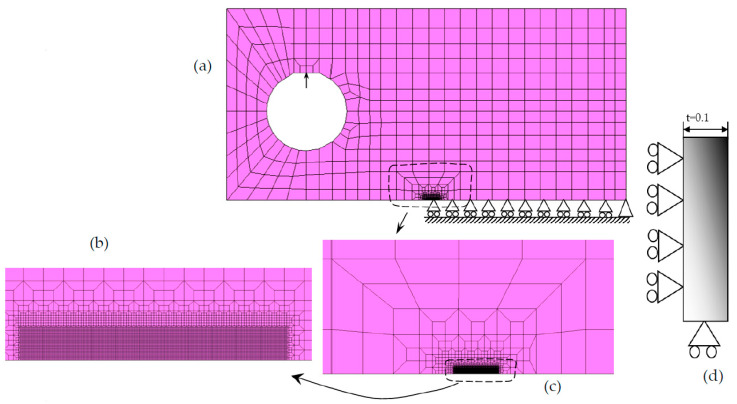
Model of C(T) specimen. (**a**,**d**) Load and boundary conditions. (**b**,**c**) Details of finite element mesh.

Figure 1a presents the geometry of the CT specimen, which has a width *W* = 50 mm. Only half of the specimen was modeled using suitable boundary conditions, as illustrated. A straight crack was defined, with the initial size, *a_0_*, of 16.5 mm. A small thickness (*t* = 0.1 mm) was considered in the numerical models, which is enough to simulate plane stress and plane strain states considering adequate boundary conditions. In the present study, a pure plane stress state was simulated, imposing displacement restrictions perpendicular to the main face of the specimen, as shown in Figure 1d. In order to take into account the physical contact between the crack flanks, a rigid surface aligned with the crack symmetry plane is considered. The specimen was submitted to a cyclic loading of constant amplitude, ranging between 41.67 N and 4.17 N, providing a stress ratio *R* = 0.1. The duration of each load cycle is 2 s. These loads give Δ*K* values of about 12 MPa.m^0.5^, which provide FCG rates in the Paris law regime [36].

### 2.2. Material Model

The accurate modelling of material elastic-plastic behavior is fundamental for the quality of numerical predictions. The elastic behavior is assumed isotropic and described by Hooke’s law. On the other hand, the rate-independent plastic behavior is characterized by the von Mises yield criterion and the Swift isotropic hardening law coupled with Lemaître–Chaboche kinematic hardening law under an associated flow rule. This kinematic hardening model can predict strain ratcheting, which is related to the progressive strain accumulation induced by the superposition of a cyclic secondary load to a constant primary load. This leads to a shift of the stress–strain hysteresis loop along the strain axis. Swift hardening law is described by [40]:

(1) $$Y=C{\left[{\left(\frac{Y_{0}}{C}\right)}^{\frac{1}{n}}+\;{\bar{\varepsilon }}^{p}\right]}^{n}$$

where *Y*_0_, *C*, and n are the material parameters and ${\bar{\varepsilon }}^{p}$ denotes the equivalent plastic strain. The Lemaître–Chaboche kinematic hardening law is [41]:

(2) $$\dot{X}{=C}_{x}\left[X_{\mathrm{Sat}}\frac{\sigma ’-X}{\bar{\sigma }}-X\;\right]{\overset{\cdot }{\bar{\varepsilon }}}^{p}$$

where *C_x_* and *X_Sat_* are the material parameters of Lemaître–Chaboche law, $\bar{\sigma }$ is the equivalent stress, and ${\overset{\cdot }{\bar{\varepsilon }}}^{p}$ is the equivalent plastic strain rate. Since the plastic constitutive model is rate-independent, the variable time is only relevant in the thermal problem for defining the temperature distribution and consequent thermal residual stresses. The calibration of the material parameters that best describe the plastic behavior was made out by minimizing the following least squares cost function:

(3) $$F(A)=\overset{N}{\underset{i=1}{\sum }}{\left(\frac{\sigma^{\mathrm{Fit}}{(A)-\sigma }^{\mathrm{Exp}}}{\sigma^{\mathrm{Exp}}}\right)}_{i}^{2},$$

where $\sigma^{\mathrm{Fit}}\left(A\right)$ and $\sigma^{\mathrm{Exp}}$ denote the fitted and the experimental values of true stress, respectively. Table 1 presents the set of numerical parameters used to describe the 2024-T351 aluminum alloy [36].

### 2.3. Generation of Residual Stresses

Two approaches were followed to generate the residual stresses. In the first approach the residual stresses are perfectly elastic, while in the second approach they result from plastic deformation.

#### 2.3.1. Case A: Elastic Residual Stresses

The region of heating by convection is defined by a rectangular zone with dimensions 300 × 210 μm^2^, which is located at 1.35 mm ahead of the initial crack front in a single face of the specimen. Two different values of convection coefficient are adopted in this study, namely *h* = 10 kW/(m^2^K) in the first load cycle and *h* = 1 kW/(m^2^K) in the subsequent load cycles. This allows the quick heating of this zone of the specimen during the first load cycle, providing a temperature rise of about Δ*T* = 50 °C, which is kept constant during the entire loading phase to provide a constant thermal residual stresses field. The temperature field is maintained constant by avoiding dissipation of the heat by conduction within the specimen. Thus, the thermal conductivity is assumed very low in the xOy plane (*k* = 0.01 W/(mK)), which inhibits the thermal fluxes in this plane and, thus, maintains the imposed temperature field. On the other hand, the thermal conductivity adopted in the thickness direction is the value typical of the aluminum alloys, *k* = 200 W/(mK), which guarantees a uniform temperature field in this direction. The thermal residual stresses arise due to the thermal expansion of the materials. The idea is to create thermal residual stresses mainly in the loading direction. Hence, the thermal expansion coefficient is considered non-null only in the y direction (α = 1.0 × 10^−4^ 1/K), producing compressive thermal residual stresses in this direction when the temperature rises. In summary, heating the material with a low conductivity creates residual stresses in a controlled manner which, together with the removal of the contact, helps to identify the mechanism responsible for the effect of residual stresses.

#### 2.3.2. Case B: Residual Stresses under Plastic Deformation

The region of cooling–heating by convection is identical to the one defined in case A, using *h* = 10 kW/(m^2^K) for the convection coefficient. The cooling of this zone of the specimen provides a temperature decrease of about Δ*T* = 180 °C, which generates localized plastic deformation due to the tensile thermal residual stresses. Two different values of thermal expansion coefficient are adopted in the y direction, namely α = 1.5 × 10^−4^ 1/K for a small level of plastic deformation (1.2%) and α = 3.0 × 10^−4^ 1/K for a high level of plastic deformation (4.6%). Then, the heating of this zone is carried out using the same convection coefficient value to achieve the initial temperature, which is kept constant during the entire loading phase to provide a constant thermal residual stresses field. The objective of this cooling stage followed by a heating stage is to produce both compressive thermal residual stresses in the loading direction and plastic deformation. 

### 2.4. Finite Element Model

The finite element mesh of the (half) specimen is composed by 7287 linear hexahedral finite elements and 14,918 nodes. The finite element mesh, shown in Figure 1b,c, was refined in the crack growth region (comprising the heating region), having 8 × 8μm^2^ of the element size. Only one layer of elements was considered along the thickness direction. Crack propagation was numerically modeled by the successive debonding of both current crack front nodes at a minimum load. The adopted fatigue crack growth criterion is based on cumulative plastic strain at the crack tip [29]. The nodal release occurs when the cumulative plastic strain at the crack tip during the entire cyclic loading reaches a critical value, assuming that cyclic plastic deformation is the crack driving force. Therefore, only one material parameter is required for this FCG criterion, which simplifies its usage. The critical value of accumulated plastic strain was calibrated for this aluminum alloy in a previous work, comparing experimental *da/dN* values with numerical predictions [37]. The numerical values of *da/dN* are obtained by dividing the crack increment of 8 μm (element size) by the number of load cycles required to reach the critical value of total plastic strain. The approach is robust with respect to mesh changes because the decrease in the size of the crack tip elements is accompanied by a decrease in the number of load cycles required to propagate the crack.

### 2.5. Software

The numerical simulations were performed using the in-house three-dimensional thermo-elastic-plastic finite element code DD3IMP (Version 1.8, University of Coimbra, Coimbra, Portugal) [42]. An updated Lagrangian approach is used to describe the evolution of the deformation process, assuming a hypoelastic-plastic model. The numerical model considers large elastoplastic strains and rotations, while the elastic strains are assumed negligibly small. The thermomechanical coupling is carried out through a staggered algorithm [43]. Nevertheless, in the present study, the coupling is assumed unidirectional, i.e., the mechanical behavior is independent of the thermal solution. Therefore, the applied temperature field only produces residual stresses by means of the material thermal expansion, keeping the mechanical behavior of the material independent of the temperature, which simplifies the FCG analysis. Indeed, the only purpose of the heating/cooling is to generate residual stresses ahead of the crack front. The thermal transient analysis is solved using the Euler’s backward method. The heat exchange to the environment is defined by convection, which helps to define the local heating of the specimen.

## 3. Results

### 3.1. Residual Stresses

**Figure 2:**
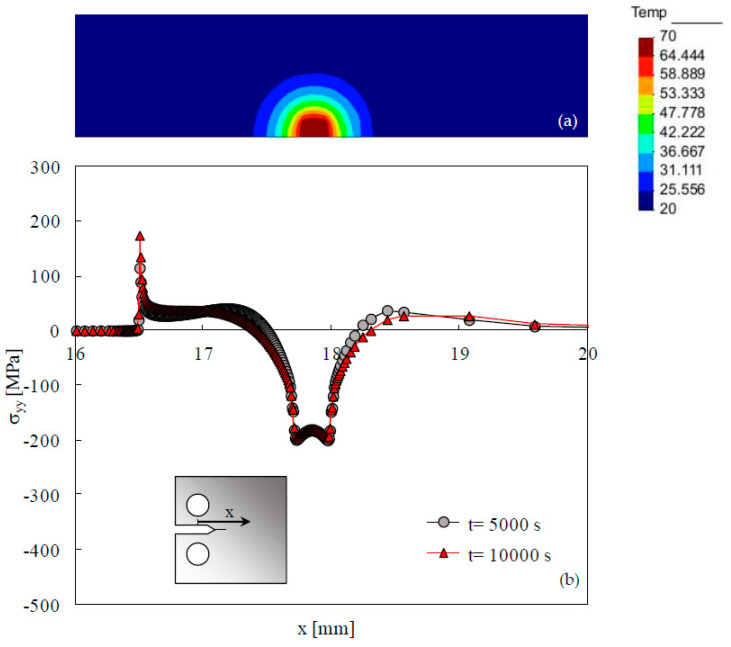
(**a**) Temperature field for t = 10,000 s. (**b**) Distribution of thermal residual stresses in the elastic regime at the symmetry line for two different instants (*a* = *a*_0_ = 16.5 mm).

Figure 2a plots the temperature field predicted by the thermal model for the instant *t* = 10,000 s. This instant corresponds to an application of 5000 load cycles when the crack propagation is considered. The initial temperature was assumed to be 20 °C, while the temperature used for the thermal convection was 70 °C. The temperature rise of 50 °C expands the material in the location of heating. The surrounding material resists to this expansion, producing elastic compressive stresses in the heating region. Figure 2b plots the thermal residual stresses at the symmetry line of the CT specimen, comparing two different instants of time, namely *t* = 5000 s and *t* = 10,000 s. The curves are almost overlapped, indicating that the very low heat conduction within the material does not significantly change the temperature profile and, consequently, the elastic residual stresses distribution. This issue is important because a high value of heat conduction would dissipate the thermal stresses during the time required for crack growth. There is a plateau of compressive residual stress ranging from −180 to −200 MPa, at the region corresponding to the maximum temperature rise. Tensile stresses are observed on both sides of these minimum values, which could be expected since an internal balance of stresses must exist. The transition between the compressive and the tensile stresses is relatively sudden. At the crack tip, i.e., for x = 16.5 mm, there is a peak of tensile stresses which opens the crack. Note that these thermal residual stresses are elastic and that the maximum compressive stress (200 MPa) is 69% of material’s yield stress (288.96 MPa). Keller et al. [44] considered residual stresses, representing 0.85 of yield stress in 2024 aluminum alloy. Hu et al. [45] also used a temperature increase to generate residual stresses. Ray et al. [46] heated a spot ahead of crack tip using a 12 mm diameter steel button.

**Figure 3:**
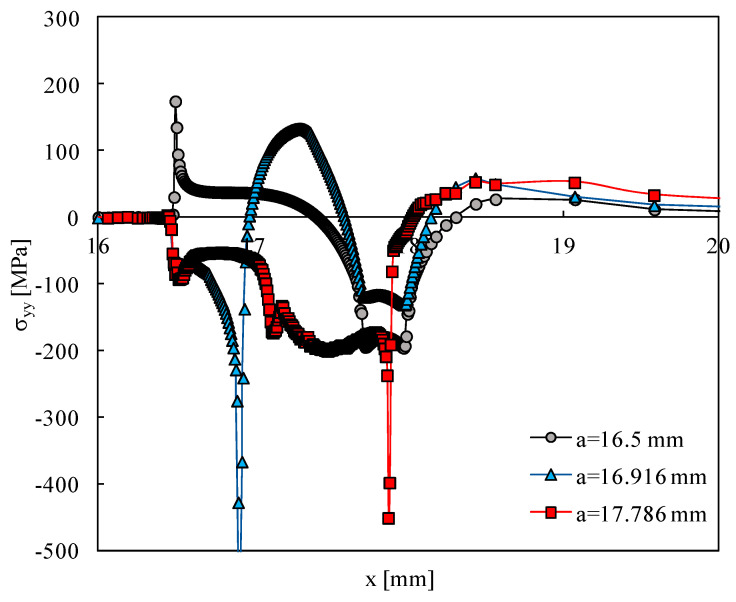
Effect of crack propagation on residual stresses.

Figure 3 plots the effect of crack propagation on the profile of residual stresses for three crack lengths. For the initial crack length (*a* = 16.5 mm), there is a peak of tensile residual stresses at the crack tip because there are no residual stresses induced by plasticity. The crack propagation significantly changes the distribution of elastic residual stresses. For both *a* = 16.916 mm and *a* = 17.786 mm, there is a peak of compressive stresses immediately ahead of the crack tip, resulting from crack tip plastic deformation. Therefore, the plastic deformation-induced residual stresses add to the thermally induced elastic stresses. For *a* = 16.916 mm, the compressive region produced by the temperature field still is well defined. However, for *a* = 17.786 mm, this region disappeared, and the thermal compressive stresses are felt behind the crack tip.

**Figure 4:**
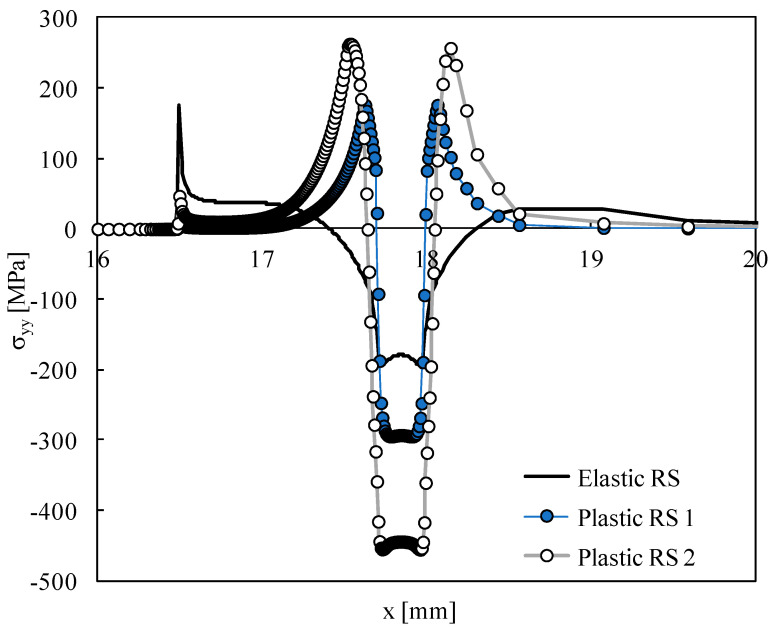
Distribution of residual stresses induced by plastic deformation.

Figure 4 plots the profiles of residual stresses obtained with the plastic deformation. The region with compressive residual stresses has a size similar to that observed for the elastic stresses; however, the magnitude of stresses is higher due to the plastic deformation. Tensile stresses are observed on both sides of the compressive region, occupying an extension of about 0.5 mm. The transition from compressive to tensile stresses is very fast. The increase in the thermal expansion coefficient adopted in the thermal process significantly increased the magnitude of maximum tensile and compressive stresses. A similar residual stress profile was obtained by Troiani et al. [9] using laser shot peening (LSP). Tensile residual stresses were also observed in both sides of the LSP pattern in order to restore the global stress equilibrium of the panel.

### 3.2. Fatigue Crack Growth Rate

**Figure 5:**
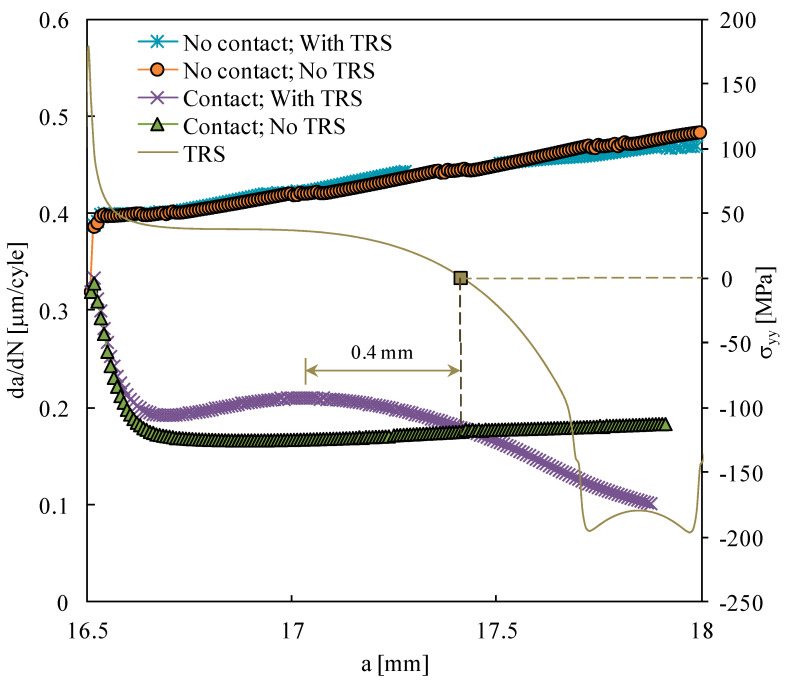
Fatigue crack growth rate versus the crack length for the elastic residual stresses (case A).

Figure 5 plots the FCG rate predicted numerically when the residual stresses are perfectly elastic. Assuming contact of the crack flanks and no thermal residual stresses (TRS), there is a decrease in *da/dN* at the beginning of crack propagation, due to the formation of residual plastic wake. After stabilization, there is a slow and progressive increase in *da/dN* with crack propagation, explained by the increase in crack tip stresses and strains with crack length. The inclusion of TRS, keeping the contact of crack flanks, changes the FCG rate. After the initial transient regime, which is similar to that observed without TRS, there is an increase in *da/dN* followed by a decrease in the minimum value. The comparison with the distribution of TRS, which is also presented in Figure 5, indicates that there is in increase in *da/dN* in the region of tensile stresses, relative to the situation without TRS. The peak corresponds to an increase of 25% in *da/dN*, which is produced by tensile stresses of 37 MPa. When the crack tip enters the region affected by compressive residual stresses, there is a progressive decrease in the FCG rate. The decrease in *da/dN* starts when the crack tip plastic zone, with a size of 0.4 mm, enters the compressive region. The crack tip position, corresponding to the transition from tensile to compressive TRS, is also the intersection of the curves, corresponding to the situations with and without TRS. In other words, the variations of *da/dN* respect the trends of residual stresses observed in Figure 2, and the changes observed in Figure 3 with crack growth do not affect the FCG rate. Bueckner [47] also suggested that, for linear elastic materials, the redistribution of applied and residual stresses due to FCG is of no consequence when computing stress intensity factors and subsequent FCG. Troiani et al. [9] used laser shot peening to treat straight patterns, 10 mm wide and 100 mm long, located ahead of the crack tip. They also obtained an increase in *da/dN* in the tensile region and a decrease in the compressive region.

The elimination of the contact of crack flanks, i.e., of crack closure, significantly changes the trends observed. The values of *da/dN* are now significantly higher, which is explained by the inexistence of crack closure effects. The transient effect observed at the beginning of crack propagation also disappears, once again because of the absence of crack closure. The increase in the FCG rate with the crack length is now more pronounced than observed with crack closure (and no TRS). A similar trend was observed in the propagation of cracks from notches [48]. However, more importantly, the results with TRS are now coincident with the predictions obtained without TRS. This indicates that the influence of TRS is linked to crack closure effects. Similarly, the elimination of the contact of crack flanks also showed that the influence of overloads is linked to crack closure effects [22].

**Figure 6:**
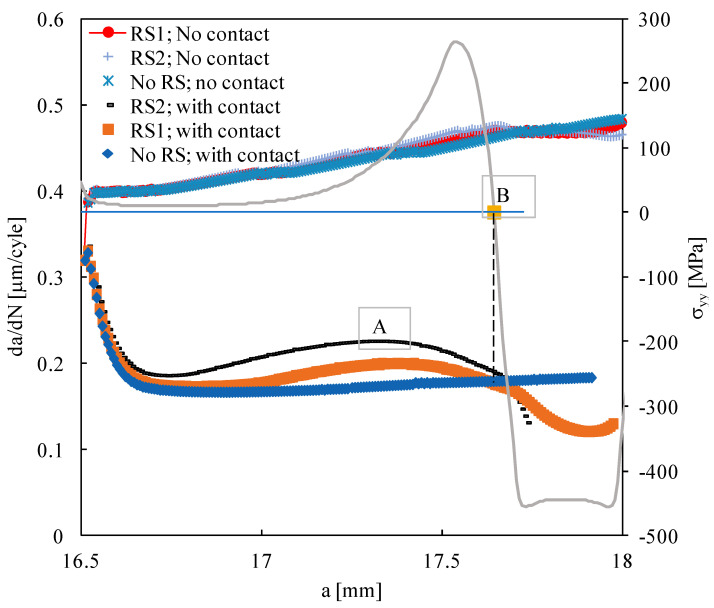
Fatigue crack growth rate versus the crack length for the residual stresses induced by plastic deformation (case B).

Figure 6 plots *da/dN* versus the crack length for the situations involving residual stresses induced by plastic deformation (case B). The global trends are similar to those obtained for the elastic residual stresses (case A). With contact of crack flanks, i.e., with crack closure, the compressive RS decrease *da/dN* relatively to the situation without RS, while the tensile RS increase *da/dN*. The maximum increase in *da/dN*, at point *A*, is 30%. Point *B*, defining the transition from tensile to compressive stresses, also defines the intersections between the curves of *da/dN*, corresponding to situations with and without RS, as indicated by the vertical dashed line. The numerical elimination of the contact of crack flanks increases significantly *da/dN*. Moreover, the effect of RS on *da/dN* almost disappears, indicating, once again, that crack closure is the mechanism behind the effect of RS.

### 3.3. Crack Closure

**Figure 7:**
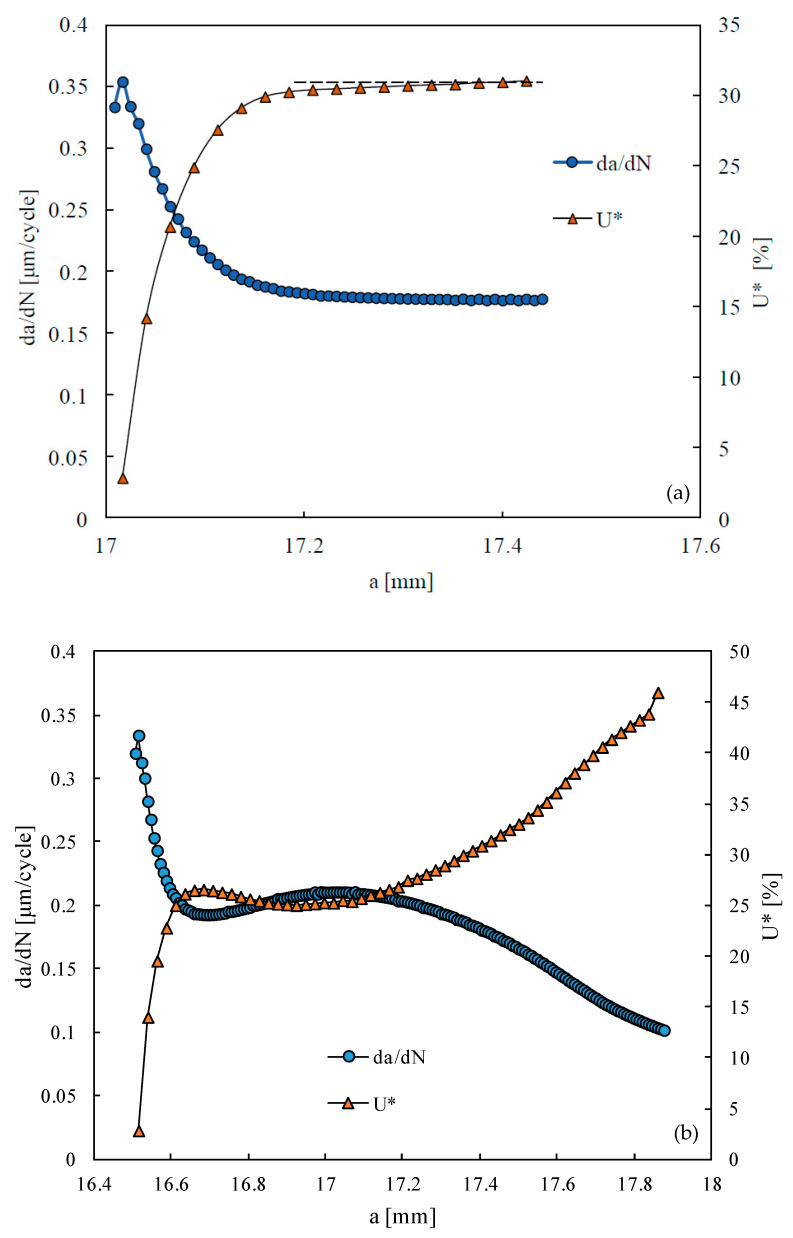
Crack closure level versus crack length in case A. (**a**) Without TRS. (**b**) With TRS.

Figure 7 presents the crack closure level, quantified by:

(4) $$U^{*}=\frac{F_{\mathrm{open}}{-F}_{\min}}{F_{\max}{-F}_{\min}}\;\times \;100,$$

where *F_open_* is the crack opening load, and *F_min_* and *F_max_* are the minimum and maximum loads, respectively. The crack opening load was defined from the contact status of the first node behind the crack tip. Figure 7a show the results without TRS, and a fast increase in U* is observed at the beginning of crack growth, as a result of the formation of residual plastic wake. After this transient behavior, which occupies nearly 140 μm, there is a slight increase in U* with crack length. The horizontal dashed line is used as reference. This behavior is typical of FCG under constant amplitude loading [49]. In the situation with TRS (Figure 7b), there is also a transient behavior of U* at the beginning of crack growth, similar to that observed in Figure 7a. After that, U* is not constant, with a slight decrease in U* followed by a significant increase. The comparison of Figure 7a,b indicates that the residual stresses significantly affect the crack closure level. Besides, in both figures, the variation of U* is perfectly symmetric to the variation of *da/dN*. This indicates that there is a link between the variations of crack closure and the trends of *da/dN*.

**Figure 8:**
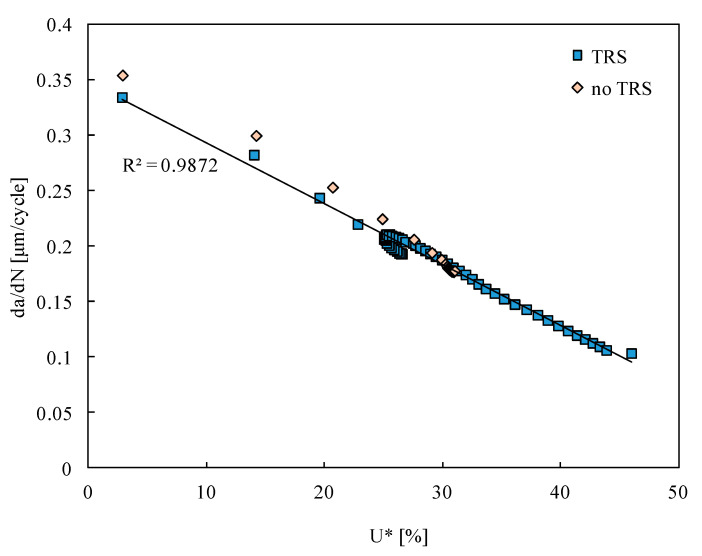
FCG rate versus crack closure level quantified by U*.

Figure 8 plots the variation of the FCG rate with the crack closure level. As expected, the increase in U* decreases *da/dN* because the effective load range is reduced. Furthermore, there is an interesting linear relation between U* and *da/dN*. The link of the effect of residual stresses on *da/dN* to variations of crack closure was mentioned by other authors. According to Lacarac [10], it is generally accepted that reduced crack growth rate is the result of increased crack closure due to the presence of compressive residual stresses. LaRue and Daniewicz [15] showed that the predicted crack closure levels increase significantly with residual stresses produced by overloading of a hole. The presence of an initial residual stress field may cause significant differences in the crack closure behavior when compared to an analysis containing no initial residual stress. Sun et al. [50] also concluded that the deceleration of FCG due to the compressive residual stress was caused by the crack closure effect. Farrahi et al. [6] showed that crack closure depends on the residual stress field. The experimental observations showed that the closure effects were stronger in the case of peening process behind the crack tip [43]. Therefore, Farrahi et al. [6] applied shot peening behind the crack tip. Crack closure seemed to account for the crack growth behavior of the peening and indentation processes. As the crack propagates a sufficient distance from the residual stress field, the closure level for all specimens reaches a constant low value. Lacarac et al. [10] studied the effect of cold-working on FCG from holes. The decrease in fatigue crack growth in cold-expanded specimens was related to higher crack opening stresses which is a consequence of the presence of compressive residual stresses arising from cold expansion. For cracks shorter than about 3 mm, the crack opening stress for cold expanded holes appears to be affected only by residual stresses arising through cold expansion. For longer cracks, plasticity-induced closure started to play an important role. The increase in stress ratio seems to reduce the positive effect of compressive stresses. Sohel Rana et al. [12] related the decrease in FCG in cold-expanded specimens to higher crack-opening stresses which are a consequence of the presence of compressive residual stresses arising from cold expansion.

### 3.4. CTOD Plots

**Figure 9:**
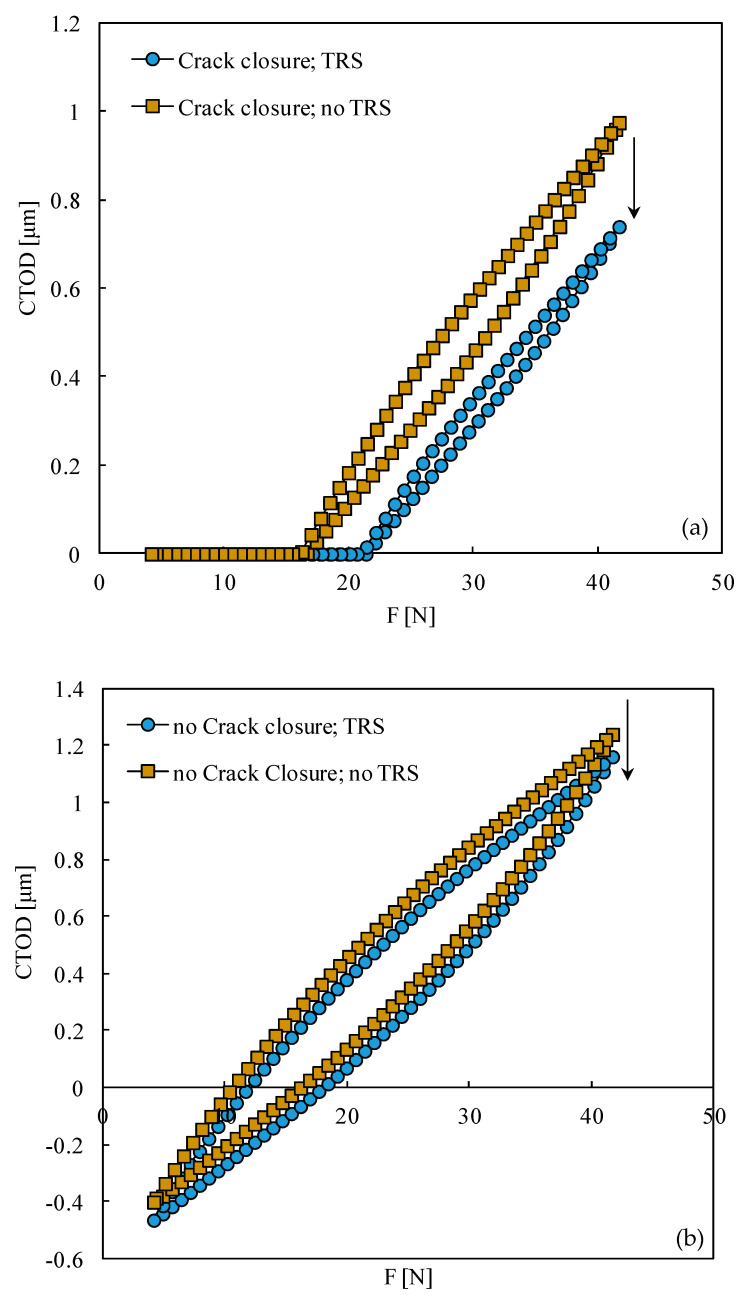
Crack tip opening displacement (CTOD). (**a**) With contact of crack flanks. (**b**) Without contact of crack flanks (a = 17.876 mm).

Figure 9 plots the crack tip opening displacement (CTOD) measured at the first node behind the crack tip, at a distance of 8 μm. The crack length was a = 17.876 mm; therefore, the crack tip is in a region affected by compressive residual stresses, as can be seen in Figure 2 and Figure 4. These compressive residual stresses move the CTOD curve down. With contact of crack flanks (Figure 9a), this movement produces more crack closure and, therefore, reduces the effective load range, the crack tip plastic deformation, and *da/dN*. Without crack closure (Figure 9b), the vertical movement of the curve does not affect the shape of the curve, so it does not affect cyclic plastic deformation at the crack tip. There is only a change in the crack tip stress ratio. In other words, assuming that cyclic plastic deformation is the main mechanism behind FCG, the compressive residual stresses do not affect directly FCG.

## 4. Conclusions

The main conclusions are:

- Elastic residual stresses (case A) were produced ahead of the crack tip by heating a rectangular region of the specimen up to 70 °C. The maximum compressive stress (200 MPa) is 69% of material’s yield stress. Tensile residual stresses are observed on both sides of the compressive region, which is a consequence of the internal balance of stresses. Residual stresses were also produced by plastic deformation (case B). In this case, the material was cooled to generate plastic deformation and then heated up to the initial temperature to create tensile residual stresses. The maximum tensile and compressive residual stresses are now bigger and the transition from compressive to tensile residual stresses is very sharp.
- The fatigue crack growth changes the distribution of elastic thermal residual stresses (TRS). The residual stresses resulting from the crack tip plastic deformation add to the TRS.
- The regions with tensile TRS show an increase in *da/dN*, while the regions with compressive TRS show a decrease in *da/dN*. A perfect match exists between the trends of *da/dN* and the original profile of TRS.
- The removal of the contact of crack flanks increased FCG rate and, more importantly, eliminated the influence of TRS. This clearly indicates that the TRS affect *da/dN* indirectly, through plasticity-induced crack closure.
- No substantial difference was found between the effects of elastic residual stresses and the residual stresses induced by plastic deformation.
- A perfect match was found between the trends of *da/dN* and crack closure, which reinforced the conviction that crack closure is responsible for the effect of TRS on the FCG rate.
- The analysis of CTOD showed that without contact of crack flanks, the residual stresses change the vertical position of CTOD versus load loop, without changing the crack tip plastic deformation.
- The numerical predictions were obtained by assuming that crack tip plastic deformation is the crack driving force. The residual stresses may directly affect other damage mechanisms of FCG, namely the growth of microvoids, environmental damage, and brittle failure.

## Figures and Tables

**Table 1:** List of material parameters involved in the Swift and Lemaître–Chaboche laws.

Material	*Y*_0_ [MPa]	*C* [MPa]	n	*C_x_*	*X_Sat_* [MPa]
AA2024-T351	288.96	389.00	0.056	138.80	111.84

## Data Availability

Data is contained within the article.
